# Low Occurrence of Infections and Death in a Real-World Cohort of Patients with Cardiac Implantable Electronic Devices

**DOI:** 10.3390/jcm12072599

**Published:** 2023-03-30

**Authors:** Jacopo Francesco Imberti, Davide Antonio Mei, Riccardo Fontanesi, Luigi Gerra, Niccolò Bonini, Marco Vitolo, Vincenzo Turco, Edoardo Casali, Giuseppe Boriani

**Affiliations:** 1 Cardiology Division, Department of Biomedical, Metabolic and Neural Sciences, University of Modena and Reggio Emilia, Policlinico di Modena, 41124 Modena, Italy; 2 Clinical and Experimental Medicine PhD Program, University of Modena and Reggio Emilia, 41125 Modena, Italy

**Keywords:** cardiac implantable electronic devices, cardiac resynchronization therapy, endocarditis, implantable cardioverter defibrillator, infection, pacemaker

## Abstract

Background. The incidence of infections and death in patients implanted with cardiac implantable electronic devices (CIEDs) is not fully known yet. Aim. To describe the incidence of CIED-related infection and death, and their potential predictors in a contemporary cohort of CIED patients. Methods. All consecutive patients implanted with a CIED at our institution were prospectively enrolled. Follow-up visits were performed 2 weeks after CIED implantation for all patients, and then every 6 months for implantable cardioverter defibrillator (ICD)/cardiac resynchronization therapy (CRT) patients and every 12 months for pacemaker (PM) patients. The adjudication of CIED-related infections was performed by two independent investigators and potential disagreement was resolved by a senior investigator. Results. Between September 2016 and August 2020, a total of 838 patients were enrolled (34.6% female; median age 77 (69.6–83.6); median PADIT score 2 (2–4)). PMs were implanted in 569 (68%) patients and ICD/CRT in 269 (32%) patients. All patients had pre-implant antibiotic prophylaxis and 5.5% had an antibiotic-eluting envelope. Follow-up data were available for 832 (99.2%) patients. After a median follow-up of 42.3 (30.2–56.4) months, five (0.6%) patients had a CIED-related infection and 212 (25.5%) patients died. Using multivariate Cox regression analysis, end-stage chronic kidney disease (CKD) requiring dialysis and therapy with corticosteroids was independently associated with a higher risk of infection (hazard ratio (HR): 14.20; 95% confidence interval (CI) 1.48–136.62 and HR: 14.71; 95% CI 1.53–141.53, respectively). Age (HR: 1.07; 95% CI 1.05–1.09), end-stage CKD requiring dialysis (HR: 6.13; 95% CI 3.38–11.13) and history of atrial fibrillation (HR: 1.47; 95% CI 1.12–1.94) were independently associated with all-cause death. Conclusions. In a contemporary cohort of CIED patients, mortality was substantially high and associated with clinical factors depicting a population at risk. On the other hand, the incidence of CIED-related infections was low.

## 1. Introduction

In the last two decades, the implant rate of cardiac implantable electronic devices (CIEDs) has considerably grown due to an aging population and improved patient characterization [1,2,3,4,5]. As a consequence, the absolute number of CIED-related complications has increased as well. CIED-related infections are among the most dangerous complications and are associated with substantial morbidity and mortality, carrying high costs for healthcare systems [6,7].

The actual incidence and prevalence of CIED-related infections is difficult to estimate, as most studies in the literature adopt different nonhomogeneous definitions of infection, antibiotic prophylaxis, surgical techniques and patient populations [8]. In a recent study including 97,750 patients, the incidence of CIED-related infection was 2.04/1000 device-years after the first implant [9]. Greenspon et al. observed that the incidence in the USA increased from around 1.5% in 2004 to 2.5% in 2008 [10]. On the other hand, data derived from the PADIT [11] and WRAP-IT [12] trials have suggested that the infection rate may be lower (0.6–1.3%).

Long-term data on CIED-related infections and mortality in patients with CIEDs are few and their determinants are still poorly understood. In the present study, we aimed to describe the long-term incidence of CIED-related infections and death in a contemporary real-world cohort of CIED patients, and aimed to identify their potential predictors.

## 2. Materials and Methods

### 2.1. Study Design and Population

We performed a prospective observational single-center study enrolling consecutive patients aged 18 years or more referred to our institution for a CIED-related procedure between September 2016 and August 2020. CIED-related procedures included the following: de novo implant, pocket revision, contralateral reimplant, system upgrade, battery replacement of pacemaker (PM), implantable cardioverter defibrillator (ICD) or cardiac resynchronization therapy (CRT). Implantable loop recorders were excluded. All patients underwent pre-procedure antibiotic prophylaxis. A single 2 g dose of cefazolin (or 900 mg of clindamycin in patients allergic to beta-lactam antibiotics) was used whenever possible. A second dose was administered in the case of procedures lasting more than 3 h. Vancomycin was used in methicillin-resistant Staphylococcus-aureus-positive patients. Antibiotic doses were adjusted according to renal function if needed. Antibiotic prophylaxis in patients potentially not represented by the previously mentioned protocol was discussed with the infectious disease specialist and tailored accordingly. An antibiotic-eluting envelope was used only in ICD/CRT patients deemed at high risk of infection according to a PADIT score > 6 [13] or dialysis [14,15]. The prevention of CIED infection before, during and after the implantation procedure was performed according to current guidelines/consensus documents [8,16].

### 2.2. Data Collection and Study Outcomes

Data were obtained at enrolment and follow-up visits by expert cardiologists and stored in a prespecified anonymized database. Implant data included the following: CIED type (e.g., PM, ICD or CRT), number of leads implanted, implant type (e.g., de novo implant, system upgrade, battery replacement, system revision, contralateral reimplant, reimplant after system extraction), antibiotic prophylaxis and antibiotic-eluting envelope. Patient data included demographic information and the presence of baseline risk factors for device-related infection according to the current literature (e.g., chronic kidney disease (CKD), dialysis, diabetes, heart failure, atrial fibrillation (AF), fever 24 h before implant, in-hospital-acquired infection, previous pocket complications, use of corticosteroids, immunosuppressive agents or anticoagulants and the use of a temporary transvenous pacemaker). The PADIT risk score was calculated according to its original definition [17]; other definitions that we used are reported in Supplemental Appendix S1. Two groups of patients were identified based on the type of device: (i) patients implanted with a PM and (ii) patients implanted with an ICD or CRT. Follow-up was performed according to our institution’s protocol: the first outpatient visit was performed 2 weeks after CIED implantation. Thereafter, follow-up visits were scheduled every 6 months for ICD/CRT and every 12 months for PM patients. Follow-up data included CIED-related infections and death for any cause. The adjudication of CIED-related infections was performed by two independent investigators (R.F. and D.A.M.) and potential disagreement was resolved by a senior investigator (G.B.). CIED-related infections were classified according to the 2020 European Heart Rhythm Association (EHRA) consensus on how to prevent, diagnose and treat CIED infections [8]. Moreover, the patient’s status was also checked using a regional database collecting all out- and in-patient visits performed in the Emilia-Romagna region in order to minimize any potential underreporting of study outcomes.

The primary objective of the present study was to describe the long-term incidence of CIED-related infections and death in a contemporary real-world cohort of CIED patients. The secondary aim was to explore the potential predictors of infection and all-cause death.

### 2.3. Statistical Analysis

Continuous variables were expressed as the median with interquartile range (IQR). The Mann–Whitney U test was used for comparisons. Categorical variables were expressed as counts and percentages. For comparison, we used the chi-square test or Fisher’s exact test (if any expected cell count was less than five). Cumulative event-free survival was evaluated with the use of Kaplan–Meier estimates and results were compared with the log-rank test. Incidence rates were calculated by dividing the number of patients reaching the outcome by the total number of person-months. To evaluate the association between study variables and outcomes, a Cox univariate regression analysis was performed. Results were expressed as hazard ratio (HR), 95% confidence interval (CI) and *p*-value. Variables with a *p*-value of ≤0.05 at univariate analysis were used to build a multivariate model. A *p*-value <0.05 was considered to be statistically significant in all of the analyses. Analyses were performed using SPSS^®^ version 26 (IBM Corp, Armonk, NY, USA). The present study was approved by the local institutional review boards/ethics committee in compliance with national regulations.

## 3. Results

### 3.1. Study and Population Characteristics

**Figure 1:**
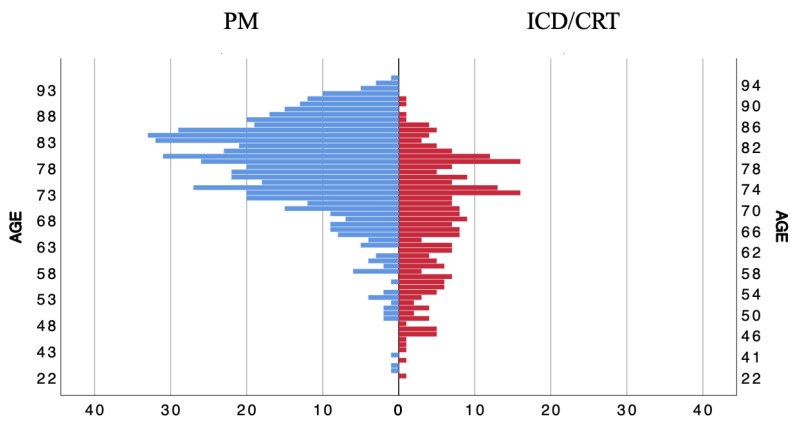
Age distribution in patients with pacemaker (PM) and implantable cardioverter defibrillator (ICD)/cardiac resynchronization therapy (CRT).

A total of 838 patients were enrolled (34.6% female; median age 77 (69.6–83.6)). A total of 658 patients (78.5%) underwent their first CIED implantation. A flow diagram of the study design is reported in Supplemental Figure S1. All patients received antibiotic prophylaxis which consisted of 2 g of cefazoline for the majority (91.6%) of them. A total of 569 patients received a PM (68%) and 269 (32%) received an ICD or CRT. PM patients were older (80 (73.6–85.2) years vs. 70 (60.8–78.0) years, *p* < 0.01) (Figure 1), and the prevalence of female sex and CKD was higher (40.9% vs. 21.2%, *p* < 0.01 and 52.5% vs. 37.9%, *p* < 0.01, respectively). On the other hand, PM patients were less likely to have a history of heart failure (5.1% vs. 61.7%, *p* < 0.01), and the overall risk of CIED-related infection according to the PADIT score was lower (2 (2–2) vs. 5 (4–7), *p* < 0.01). The two study cohorts did not differ regarding a previous history of diabetes (23.6% vs. 29.0%, *p* = 0.11), end-stage CKD requiring dialysis (2.8% vs. 2.6%, *p* = 1) and the use of corticosteroid or immunosuppressive therapy (3.0% vs. 2.2%, *p* = 0.65 and 0.9% vs. 0.0%, *p* = 0.18, respectively). The first implant procedures were more common in the PM group (87.0% vs. 60.6%, *p* < 0.01). Only three reimplantation procedures after extraction were reported and they were all performed in ICD/CRT patients. The TYRX antibiotic-eluting envelope was used only in ICD/CRT patients (46 patients; 5.5%). A detailed description of the baseline patient characteristics is reported in Table 1. The most frequent reason for implant in PM patients was high-degree atrioventricular block (48.3%), followed by sick sinus syndrome (45.3%), bradyAF (4.9%), symptomatic sinus bradycardia (0.5%) and others (1%).

### 3.2. Study Outcomes

Follow-up data were available for 832 (99.2%) patients. After a median follow-up of 42.3 (30.2–56.4) months, five (0.6%) patients had a CIED-related infection. The median age for CIED-related infection was 74.1 (70.3–78.8) years. The median time between implant/replacement and CIED-related infection was 4.4 (2.3–22.8) months. Three out of five infections occurred within six months of the index procedure, while two out of five occurred after more than 1 year. Four out of five patients required leads and generator extraction (besides antibiotics), while for one patient generator-only extraction was needed. Blood cultures were positive in two patients (methicillin-sensitive Staphylococcus aureus and Escherichia coli, respectively). None of these patients died because of the infection. The overall incidence rate of CIED-related infections was 0.1 per 1000 person-months (five first events over 35,254 person-months). Cox regression analysis showed no difference between the two study groups for this outcome (HR: 1.40; 95% CI 0.23–8.35) (Table 2). Among the tested risk factors, end-stage CKD requiring dialysis and therapy with corticosteroids was independently associated with a higher risk of infection (HR: 14.20; 95% CI 1.48–136.62 and HR: 14.71; 95% CI 1.53–141.53, respectively) (Supplemental Table S1).

**Figure 2:**
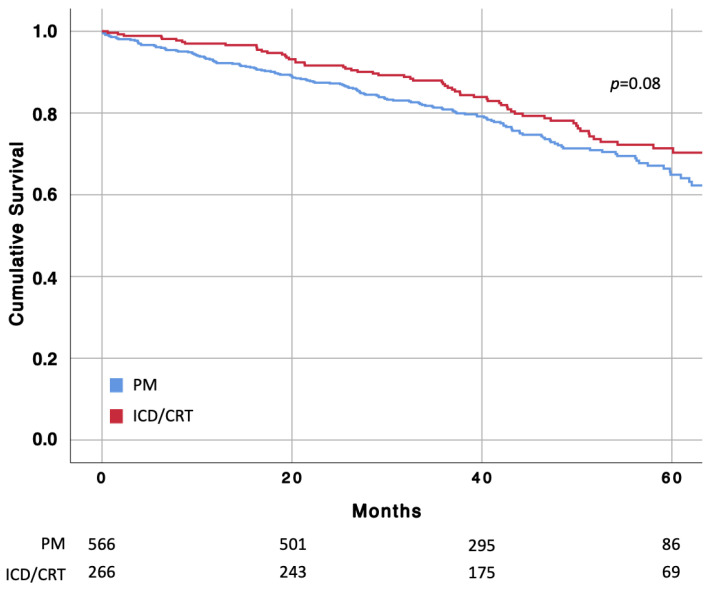
Cumulative incidence of all-cause death in patients with pacemaker (PM) and implantable cardioverter defibrillator (ICD)/cardiac resynchronization therapy (CRT) estimated using the Kaplan–Meier method.

The overall incidence rate of death was 6.0 per 1000 person-months (212 first events over 35,465 person-months). Interestingly, Kaplan–Meier analysis showed no significant difference between the two study groups (Figure 2) and this finding was also confirmed via Cox regression analysis (HR: 0.77; 95% CI 0.57–1.04) (Table 2). After adjustments, age (HR: 1.07; 95% CI 1.05–1.09), end-stage CKD requiring dialysis (HR: 6.13; 95% CI 3.38–11.13) and history of AF (HR: 1.47; 95% CI 1.12–1.94) were independently associated with all-cause death (Table 3). In the subgroup analysis, age (HR: 1.10; 95% CI 1.07–1.13) and end-stage CKD requiring dialysis (HR: 9.22; 95% CI 4.69–18.12) consistently remained the only parameters independently associated with all-cause mortality in patients with a PM (Supplemental Table S2). On the other hand, in the subgroup analysis considering only patients with ICD/CRT, age (HR: 1.04; 95% CI 1.01–1.08), CKD (HR: 2.40; 95% CI 1.25–4.62), diabetes (HR: 1.97; 95% CI 1.13–3.45), and AF (HR: 1.86; 95% CI 1.04–3.30) independently predicted all-cause mortality (Supplemental Table S3).

## 4. Discussion

Our study described the long-term incidence of CIED-related infections and death in a contemporary real-world cohort of CIED patients. Our main findings were as follows: (i) the overall incidence of CIED-related infections was substantially low (0.6%), while (ii) all-cause death was not negligible (25.5%); (iii) end-stage CKD requiring dialysis and therapy with corticosteroids were independently associated with a higher risk of infection, and (iv) age, end-stage CKD requiring dialysis and history of AF were independently associated with all-cause death.

Infections are one of the most feared complications related to CIEDs, and they are associated with significant mortality, morbidity and financial healthcare burden [18,19]. The reported 1-year mortality rate among patients with CIED-related infection undergoing transvenous lead extraction is around 20%, and its risk is almost two-fold higher in patients affected by endovascular infections as compared to patients with pocket infections [12]. In a prespecified sub-analysis of the WRAP-IT trial, Wilkoff et al. [20] showed that major CIED-related infections were associated with increased all-cause mortality and a reduced quality of life. Moreover, infections, directly and indirectly, triggered a cascade of additional healthcare-related procedures leading to higher costs. The precise incidence rate of CIED-related infections is still a matter of debate in the literature. Indeed, it varies depending on the retrospective or prospective design of studies, different patient populations, and different definitions of CIED-related infections [8]. A meta-analysis conducted in 2015 on 26,172 patients across 21 prospective studies reported an average infection rate of 1.6% [21]. Similar figures have been reported after generator replacement [22,23]. Biffi et al. [23] analyzed 983 prospectively enrolled ICD/CRT-D patients undergoing generator replacement or upgrades. After 1-year follow-up, 7% of patients died and 1.2% experienced a CIED-related infection. In a prospective study including 2675 patients across 18 centers in Italy, the investigators reported 1.1% of CIED-related infections and 5% mortality at 1-year [24]. In the context of a randomized controlled trial, the reported rate of infection was slightly lower, being 1.2% in the WRAP-IT trial [12] and 1.03% in the PADIT trial [11] at 1-year (usual care arms).

In our study, we observed less cases of CIED-related infections (0.6%) despite a long median follow-up of 3.5 years. As compared to the above-mentioned randomized controlled trials [11,12], we adopted broader inclusion criteria, reflecting a real-world scenario of unselected patients. The low incidence of CIED-related infections may be explained by strict adherence to sterile and proper surgical techniques, antibiotic prophylaxis consistently performed in all patients and high operator experience (all cases were performed by expert electrophysiologists working in a tertiary referral center). In a recent study by Olsen et al. [15], the median time to CIED infection was 296 (68–946) CIED-days, with systemic infections occurring significantly later than pocket infections. The former appeared to be associated with risk factors predisposing patients to bacteremia, while the latter was associated with surgery-related factors. In our cohort, three out of five infections occurred within six months of the index procedure, while two out of five occurred after almost 2 years. Our results reinforce and further build upon recent studies, suggesting that the risk of CIED-related infections is not only related to the procedure itself, but that it also extends throughout the patient’s lifetime [23,25] and may occur even years after a CIED-related procedure [26]. A potential practical implication aimed at improving outcomes may be to carefully plan long-term comprehensive and holistic patient management, not limited to CIED checks and oriented toward specific patient clinical factors. Tailored and patient-oriented management may reduce mortality in the complex scenario of CIED infections as well, with the evaluation of causative organisms and complications (that have been associated with a higher risk of mortality), and possibly surgical removal of the CIED (associated with lower risk) [27]. We found that end-stage CKD requiring dialysis and treatment with corticosteroids was associated with a higher risk of infection. Our results should be taken with caution as the number of events was low. However, both parameters are well-established predictors of CIED-related infections according to the current literature [17,21,28,29].

In our cohort, all patients received pre-procedure antibiotic prophylaxis. A single dose of cefazolin was the most commonly adopted strategy. A recent study showed that patients at high risk of CIED-related infection treated with a prolonged 9-day protocol of periprocedural antibiotics had a similar rate of infection as compared to low-risk patients treated with only two antibiotic administrations (1.98% vs. 1.08%, respectively, *p* = 0.32, at 250 days of follow-up) [30,31]. The antibiotic-eluting envelope in addition to antibiotic prophylaxis was associated with reduced CIED-related major infections and local CIED infections in randomized controlled trials and real-world populations at high risk of infection [12,32,33,34]. Cost-effectiveness data support its use in selected high-risk patients whereby, by reducing CIED infections, it provides value for healthcare systems [13,35]. Accordingly, we used an antibiotic-eluting envelope only in ICD/CRT patients deemed at high risk of CIED infection (46 patients). None of these patients had a CIED-related infection during follow-up.

In our cohort, 25.5% of patients died during a median follow-up of 3.5 years. Among the tested parameters, only age, history of AF and end-stage CKD requiring dialysis were independently associated with all-cause mortality. No difference was observed between PM and ICD/CRT patients. This finding may be explained by the higher burden of comorbidities of ICD/CRT patients, partially counterbalanced by their younger age. Few studies have addressed the issue of mortality in patients with a PM [36,37,38,39]. In 2004, Brunner et al. [36] reported long-term survival after PM implantation in 6505 patients enrolled in a single center. The median age was 72.1 years and 32.6% of patients were implanted due to sick sinus syndrome. The median survival was approximately 8.5 years. The authors identified several predictors of mortality, but they varied according to the decade in which the PM was implanted. Interestingly, they found that neither the index arrhythmia nor the pacing mode was associated with increased mortality when the authors analyzed patients implanted between 1991 and 2000, while they were associated when patients previously implanted (e.g., between 1971 and 2000) were considered. A study conducted in 2008 on 1627 PM recipients with AF at implantation showed that male gender, age at implantation and non-syncopal bradycardia influenced survival, but so did the decade of implantation [37]. More recently, heart failure, chronic obstructive pulmonary disease, age, syncope, insulin-dependent diabetes mellitus and male gender have been found to be independent predictors of increased mortality in a cohort of 154 PM patients implanted due to a heart block [39]. Balla et al. [40] reported a mortality rate of 31% in a cohort of 119 PM patients aged more than 70 years and prospectively followed them for 46 months. Reduced physical performance assessed using the short physical performance battery test was independently associated with death during follow-up and the composite of death and rehospitalization at 1-year. Interestingly, the authors identified physical performance as an additional risk-stratifier besides age and comorbidities, and it may represent a potential target for tailored interventions in a holistic approach to patient care [41].

In our cohort, we found that age and end-stage CKD requiring dialysis were independently associated with all-cause mortality in patients with a PM. Interestingly, implant reasons did not predict all-cause mortality (as also reported by recent studies in the literature), and it can be speculated that patient-related factors may influence outcomes and the peri-/post-procedural complication rate more than device-related factors [42]. The PADIT score was not associated with a higher risk of mortality either. These results are in line with the current literature. In 2022, Boriani et al. [24] reported that the PADIT score was significantly associated with CIED-related infections, but it was not significantly associated with the occurrence of a composite of infection and all-cause death. The authors also studied the performance of a new score called the RI-AIAC event score. This score showed a moderate to good predictive ability for the composite outcome both in the derivation and in the validation cohorts. These findings may suggest that mortality in CIED patients may be influenced by several different risk factors, as compared to factors associated with the infective risk, and may have a relationship with specific patient characteristics, which may or may not overlap with risk factors for CIED-related infections. Finally, all-cause mortality has been evaluated in several ICD/CRT trials. In this regard, our data are in line with the current literature [43,44].

In our study, the last period of patient enrolment was during the COVID-19 pandemic, which had an important impact on the activity of all centers involved in electrophysiology and device implants [45,46,47,48]. In particular, between March 2020 and February 2022, five waves of COVID-19 were observed in Italy. A significant reduction in elective CIED implants was reported among several Italian arrhythmia centers during the first wave, with a progressive and gradual return to pre-pandemic volumes during the third–fourth waves. On the other hand, emergency procedures only dropped by 10%, and recovery was observed usually during the second wave [45].

## 5. Limitations

The adjudication of CIED-related infections was not centralized. However, it was performed by two independent investigators and disagreement was resolved by a senior investigator. Moreover, a strict and consistent definition of CIED-related infection was applied, according to the 2020 EHRA consensus on how to prevent, diagnose and treat CIED infections [12].

The relatively small number of events limited the possibility to observe potential differences between study groups in terms of outcomes, and residual confounders cannot be completely ruled out. For the same reason, the predictors of CIED-related infections that we found should be regarded as hypothesis generating and the generalizability of our results may be limited. However, the present study included a real-world cohort of all-comers, thus reflecting the clinical scenarios more often encountered during everyday clinical practice. Given the pragmatic nature of our study, it was not possible to gather granular data enabling a deeper characterization of patients. In particular, it was not possible to collect detailed baseline clinical characteristics, drug treatment or cause of death, which might have enriched our analysis. Moreover, the impact of late infections, occurring at a longer follow-up time, requires further study. Although we are not a lead extraction center, we are formally linked to a lead extraction center and there is a continuous connection for every case, thus providing adequate patient data and follow-up. It cannot be completely ruled out that some patients of CIED replacements may not have been enrolled due to a short hospital stay (Day Hospital procedure) and limited laboratory tests performed. Finally, leadless PMs and subcutaneous ICDs were not included in the present study as they deserve ad hoc clinical studies in our view.

## 6. Conclusions

In a contemporary cohort of CIED patients, mortality was substantially high and associated with clinical factors, depicting a population at risk. On the other hand, the incidence of CIED-related infections was low.

## Figures and Tables

**Table 1:** Baseline characteristics of the study cohort.

	Total (n = 838)	PM (n = 569)	ICD/CRT (n = 269)	*p*-Value
Demographic and clinical characteristics				
Age, median (IQR)	77 (69.6–83.6)	80 (73.6–85.2)	70 (60.8–78.0)	<0.01
Female sex, n (%)	290 (34.6)	233 (40.9)	57 (21.2)	<0.01
Day Hospital procedure, n (%)	106 (12.6)	60 (10.5)	46 (17.1)	0.01
CKD, n (%)	401 (47.9)	299 (52.5)	102 (37.9)	<0.01
End-stage CKD in dialysis, n (%)	23 (2.7)	16 (2.8)	7 (2.6)	1.00
Diabetes, n (%)	212 (25.3)	134 (23.6)	78 (29.0)	0.11
Heart failure, n (%)	195 (23.3)	29 (5.1)	166 (61.7)	<0.01
Atrial fibrillation, n (%)	293 (35.0)	187 (32.9)	106 (39.4)	0.06
Corticosteroids, n (%)	23 (2.7)	17 (3.0)	6 (2.2)	0.65
Oral anticoagulants, n (%)	304 (36.3)	178 (31.3)	126 (46.8)	<0.01
Immunosuppressive therapy, n (%)	5 (0.6)	5 (0.9)	0 (0.0)	0.18
PADIT score, median (IQR)	2 (2–4)	2 (2–2)	5 (4–7)	<0.01
Other potential risk factors for CIED-related infection				
Fever 24 h before implant, n (%)	6 (0.7)	6 (1.1)	0 (0.0)	0.19
HAI, n (%)	23 (2.7)	14 (2.5)	9 (3.3)	0.50
Pocket complications, n (%)	15 (1.8)	8 (1.4)	7 (2.6)	0.26
Temporary pacing, n (%)	8 (1.0)	8 (1.4)	0 (0.0)	0.06
Procedure-related features				
First implant, n (%)	658 (78.5)	495 (87.0)	163 (60.6)	<0.01
Contralateral reimplant, n (%)	6 (0.7)	2 (0.4)	4 (1.5)	0.09
Upgrading, n (%)	16 (1.9)	1 (0.2)	15 (5.6)	<0.01
Revision, n (%)	2 (0.2)	1 (0.2)	1 (0.2)	0.54
New CIED reimplantation after a CIED extraction, n (%)	3 (0.4)	0 (0.0)	3 (1.1)	0.03
Generator change, n (%)	153 (18.3)	70 (12.2)	83 (31.0)	<0.01
Antibiotic prophylaxis, n (%)				
Cefazoline	768 (91.6)	521 (91.6)	247 (91.8)	1.00
Other antibiotics	70 (8.4)	48 (8.4)	22 (8.2)	1.00
Antibiotic-eluting envelope, n (%)	46 (5.5)	0 (0.0)	46 (17.1)	<0.01

CIED, cardiac implantable electronic device; CKD, chronic kidney disease; CRT, cardiac resynchronization therapy; HAI, hospital-acquired infection; ICD, implantable cardioverter defibrillator; IQR, interquartile range; PM, pacemaker.

**Table 2:** Study outcomes.

	PM		ICD/CRT		HR (95% CI)	*p*-Value
	n/N (%)	Events/1000 Patient-Months	n/N	Events/1000 Patient-Months		
Mortality	149/566 (26.3)	6.4	63/266 (23.7)	5.1	0.77 (0.57–1.04)	0.08
Infections	3/566 (0.5)	0.1	2/266 (0.8)	0.2	1.40 (0.23–8.35)	0.72

CI, confidence interval; CRT, cardiac resynchronization therapy; ICD, implantable cardioverter defibrillator; HR, hazard ratio; PM, pacemaker.

**Table 3:** Univariate and multivariate Cox’s regression analysis for all-cause mortality.

	Univariate			Multivariate		
	HR	*p*-Value	CI	HR	*p*-Value	CI
Age	1.08	<0.01	1.06–1.10	1.07	<0.01	1.05–1.09
Male sex	0.90	0.49	0.68–1.21			
Upgrading	1.20	0.68	0.50–2.92			
Revision	0.05	0.72	0.00–NA			
New CIED reimplantation after a CIED extraction	1.37	0.75	0.19–9.77			
CKD	2.92	<0.01	2.18–3.92	-	-	-
End-stage CKD in dialysis	4.39	<0.01	2.55–7.58	6.13	<0.01	3.38–11.13
Diabetes	1.40	0.02	1.04–1.87	-	-	-
Heart failure	1.19	0.26	0.88–1.61			
Atrial fibrillation	1.65	<0.01	1.26–2.16	1.47	<0.01	1.12–1.94
Antibiotic-eluting envelope	1.29	0.37	0.74–2.27			
PADIT score						
Low risk	Ref.	Ref.	Ref.			
Intermediate risk	1.11	0.68	0.67–1.84			
High risk	1.49	0.53	0.99–2.23			
ICD/CRT	0.77	0.84	0.57–1.04			

CKD, chronic kidney disease; CRT, cardiac resynchronization therapy; HR, hazard ratio; ICD, implantable cardioverter defibrillator; NA, not applicable.

## Data Availability

The data presented in this study are available upon reasonable request to the corresponding author.

## Supplementary Materials

The following supporting information can be downloaded at https://www.mdpi.com/article/10.3390/jcm12072599/s1: Figure S1: Flow diagram of study design; Table S1: Univariate and multivariate Cox’s regression analysis for infection in the overall cohort; Table S2: Univariate and multivariate Cox’s regression analysis for all-cause mortality in patients with pacemaker; Table S3: Univariate and multivariate Cox’s regression analysis for all-cause mortality in patients with ICD/CRT. References [49,50,51,52] are cited in the supplementary materials.
