# Supplementary material for: Low Occurrence of Infections and Death in a Real-World Cohort of Patients with Cardiac Implantable Electronic Devices
Source: J Clin Med. 2023 Mar 30;12(7):2599. doi: 10.3390/jcm12072599 (PMC10095352; doi:10.3390/jcm12072599)
Supplement: Supplementary file 1 [file jcm-12-02599-s001.zip › jcm-2226033-supplementary.pdf]

SUPPLEMENTAL MATERIAL

Low occurrence of infections and death in a real-world cohort of patients with cardiac implantable electronic devices

Jacopo Francesco Imberti[1,2*], Davide Antonio Mei[1*], Riccardo Fontanesi[1], Luigi Gerra[1], Niccolò Bonini[1,2], Marco Vitolo[1,2], Vincenzo Turco[1], Edoardo Casali[1], Giuseppe Boriani[1]

[1]Cardiology Division, Department of Biomedical, Metabolic and Neural Sciences, University of Modena and Reggio Emilia, Policlinico di Modena, Modena, Italy.
[2]Clinical and Experimental Medicine PhD Program, University of Modena and Reggio Emilia, Modena, Italy

INDEX

Supplemental Figure S1. Flow diagram of study design.

Supplemental Table S1. Univariate and multivariate Cox's regression analysis for infection in the overall cohort.

Supplemental Table S2. Univariate and multivariate Cox's regression analysis for all-cause mortality in patients with pacemaker.

Supplemental Table S3. Univariate and multivariate Cox's regression analysis for all-cause mortality in patients with implantable cardioverter defibrillator (ICD)/cardiac resynchronization therapy (CRT)

Supplemental Appendix S1. Definitions.

Supplemental Figure S1. Flow diagram of study design. CIED, cardiac implantable electronic devices; CRT, cardiac resynchronization therapy; ICD, implantable cardioverter defibrillator; PM, pacemaker; pts, patients.

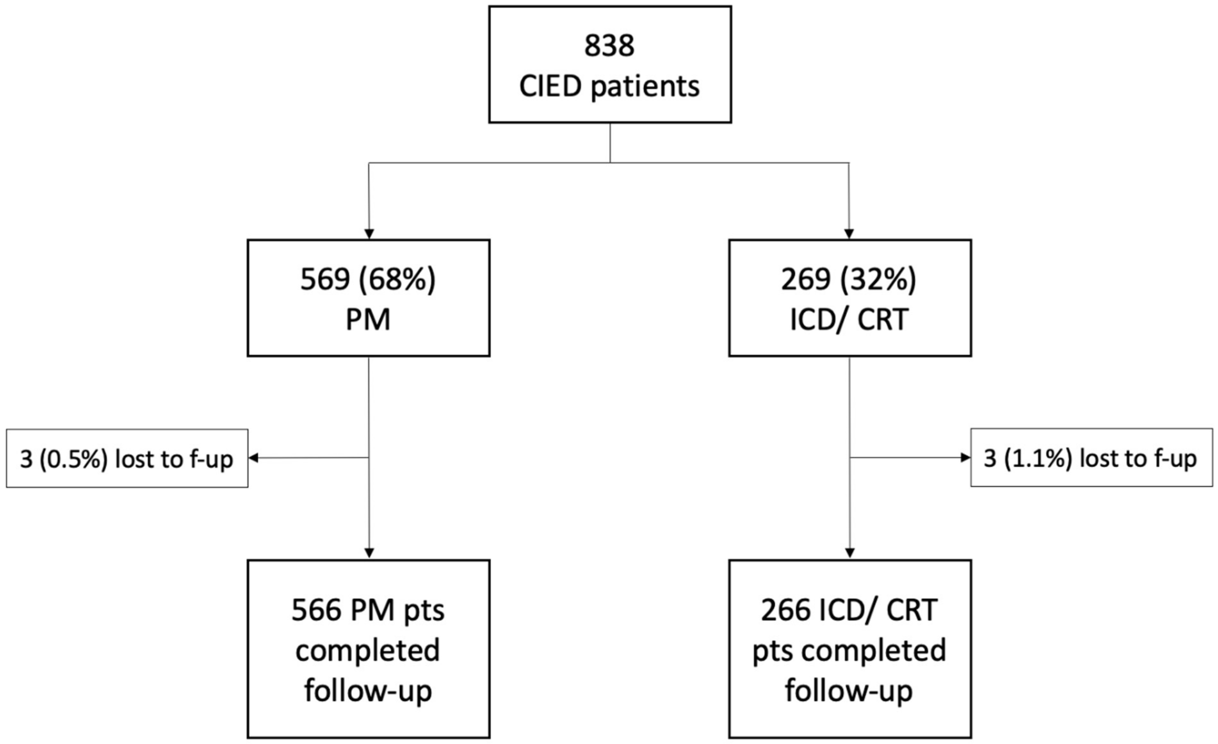

Supplemental Table S1. Univariate and multivariate Cox's regression analysis for infection in the overall cohort.

	Univariate			Multivariate		
	**HR**	**p-Value**	**CI**	**HR**	**p-Value**	**CI**
Age	-	-	-			
Male sex	-	-	-			
First implant	-	-	-			
Upgrading	-	-	-			
Revision	-	-	-			
New CIED reimplant after a CIED extraction	-	-	-			
> 2 leads implanted	-	-	-			
CKD	-	-	-			
End-stage CKD in dialysis	11.28	0.03	1.26-101.01	14.20	0.02	1.48-136.62
Diabetes	-	-	-			
Heart failure	-	-	-			
Atrial fibrillation	-	-	-			
Fever 24 hours before implant	-	-	-			
HAI	-	-	-			
Antibiotic-eluting envelope	-	-	-			
PADIT score						
Low risk	-	-	-			
Intermediate risk	-	-	-			
High risk	-	-	-			
ICD/CRT	-	-	-			
Corticosteroids	10.88	0.03	1.22-97.43	14.71	0.02	1.53-141.53
Anticoagulants	-	-	-			
Immunosuppressive therapy	-	-	-			

CI, confidence interval; CKD, chronic kidney disease; CRT, cardiac resynchronization therapy; HAI, hospital-acquired infection; HR, hazard ratio; ICD, implantable cardioverter defibrillator.

Supplemental Table S2. Univariate and multivariate Cox's regression analysis for all-cause mortality in patients with pacemaker.

	Univariate			Multivariate		
	**HR**	**p-Value**	**CI**	**HR**	**p-Value**	**CI**
Age	1.09	<0.01	1.07-1.12	1.10	<0.01	1.07-1.13
Male sex	1.01	0.94	0.73-1.41			
Upgrading	0.05	0.78	0.0-NA			
Revision	0.05	0.78	0.0-NA			
New CIED reimplant after a CIED extraction	-	-	-			
CKD	2.58	<0.01	1.80-3.69	-	-	-
End-stage CKD in dialysis	5.45	<0.01	2.93-10.13	9.22	<0.01	4.69-18.12
Diabetes	1.15	0.46	0.79-1.67			
Heart failure	1.93	0.03	1.07-3.48	-	-	-
Atrial fibrillation	1.43	0.03	1.03-1.99	-	-	-
PM indication						
AVB	Ref.	Ref.	Ref.			
SSS	0.01	0.39	0-251			
BradyAF or symptomatic sinus bradycardia	0.01	0.77	0-NA			
Antibiotic-eluting envelope	-	-	-			
PADIT score						
Low risk	Ref.	Ref.	Ref.			
Intermediate risk	1.37	0.50	0.56-3.33			
High risk	3.33	0.09	0.82-13.48			

AF, atrial fibrillation; AVB, atrioventricular block; CI, confidence interval; CKD, chronic kidney disease; CRT, cardiac resynchronization therapy; HR, hazard ratio; ICD, implantable cardioverter defibrillator; NA, not applicable; PM, pacemaker; SSS, sick sinus syndrome.

Supplemental Table S3. Univariate and multivariate Cox's regression analysis for all-cause mortality in patients with implantable cardioverter defibrillator (ICD)/cardiac resynchronization therapy (CRT).

	Univariate			Multivariate		
	**HR**	**p-Value**	**CI**	**HR**	**p-Value**	**CI**
Age	1.08	<0.01	1.05-1.11	1.04	0.03	1.01-1.08
Male sex	2.07	0.05	0.99-4.35			
Upgrading	1.51	0.37	0.61-3.78			
Revision	0.05	0.83	0.00-NA			
New CIED reimplant after a CIED extraction	1.71	0.60	0.24-12.83			
CKD	3.66	<0.01	2.18-6.14	2.40	0.01	1.25-4.62
End-stage CKD in dialysis	2.59	0.11	0.81-8.23			
Diabetes	2.14	<0.01	1.30-3.52	1.97	0.02	1.13-3.45
Heart failure	1.79	0.04	1.03-3.13	-	-	-
Atrial fibrillation	2.40	< 0.01	1.45-3.95	1.86	0.03	1.04-3.30
Antibiotic-eluting envelope	1.69	0.10	0.91-3.14			
PADIT score						
Low risk	Ref.	Ref.	Ref.	Ref.	Ref.	Ref.
Intermediate risk	1.75	0.14	0.84-3.67	-	-	-
High risk	2.51	<0.01	1.36-4.63	-	-	-

CI, confidence interval; HR, hazard ratio; NA, not applicable.

Supplemental Appendix S1. Definitions.

Atrial fibrillation is a supraventricular arrhythmia that we defined according to the 2016 ESC guidelines[49].

Chronic kidney disease was defined as kidney damage (structural abnormality or persistent hematuria and/or proteinuria) or glomerular filtration rate <60 mL/min/1.73 m2 for 3 months or more, irrespective of cause[50].

Diabetes was defined as HbA1C 6.5% or fasting plasma glucose 126 mg/dl (7.0 mmol/l). Fasting is defined as no caloric intake for at least 8 h, or 2 h plasma glucose 200 mg/dl (11.1mmol/l) during an oral glucose tolerance test (OGTT), or in a patient with classic symptoms of hyperglycemia, or hyperglycemic crisis with random plasma glucose 200 mg/dl (11.1 mmol/l) or treatment with oral hypoglycemic agent and/or insulin[51].

Heart failure is a clinical syndrome consisting of cardinal signs and symptoms (breathlessness, ankle swelling, pulmonary crackles, etc.) that we defined according to the 2016 ESC guidelines[52].

REFERENCES

49. Kirchhof, P.; Benussi, S.; Kotecha, D.; Ahlsson, A.; Atar, D.; Casadei, B.; Castella, M.; Diener, H.C.; Heidbuchel, H.; Hendriks, J.; et al. 2016 ESC Guidelines for the management of atrial fibrillation developed in collaboration with EACTS. *Eur Heart J* **2016**, *37*, 2893-2962, doi:10.1093/eurheartj/ehw210.
50. Levey, A.S.; Eckardt, K.U.; Tsukamoto, Y.; Levin, A.; Coresh, J.; Rossert, J.; De Zeeuw, D.; Hostetter, T.H.; Lameire, N.; Eknoyan, G. Definition and classification of chronic kidney disease: a position statement from Kidney Disease: Improving Global Outcomes (KDIGO). *Kidney Int* **2005**, *67*, 2089-2100, doi:10.1111/j.1523-1755.2005.00365.x.
51. Association, A.D. Diagnosis and classification of diabetes mellitus. *Diabetes Care* **2010**, *33 Suppl 1*, S62-69, doi:10.2337/dc10-S062.
52. Ponikowski, P.; Voors, A.A.; Anker, S.D.; Bueno, H.; Cleland, J.G.F.; Coats, A.J.S.; Falk, V.; González-Juanatey, J.R.; Harjola, V.P.; Jankowska, E.A.; et al. 2016 ESC Guidelines for the diagnosis and treatment of acute and chronic heart failure: The Task Force for the diagnosis and treatment of acute and chronic heart failure of the European Society of Cardiology (ESC)Developed with the special contribution of the Heart Failure Association (HFA) of the ESC. *Eur Heart J* **2016**, *37*, 2129-2200, doi:10.1093/eurheartj/ehw128.
